# The Prominin-1-Derived Peptide Improves Cardiac Function Following Ischemia

**DOI:** 10.3390/ijms22105169

**Published:** 2021-05-13

**Authors:** Avner Adini, Irit Adini, Etty Grad, Yuval Tal, Haim D. Danenberg, Peter M. Kang, Benjamin D. Matthews, Robert J. D’Amato

**Affiliations:** 1 Vascular Biology Program, Department of Surgery, Boston Children’s Hospital, Harvard Medical School, Boston, MA 02115, USA; Benjamin.matthews@childrens.harvard.edu (B.D.M.); robert.damato@childrens.harvard.edu (R.J.D.); 2 Department of Medicine, Boston Children’s Hospital, Harvard Medical School, Boston, MA 02115, USA; 3 Department of Surgery, Harvard Medical School, The Center for Engineering in Medicine, Mass General Hospital, Shriners Hospitals for Children Boston, Boston, MA 02114, USA; IADINI@mgh.harvard.edu; 4 Interventional Cardiology, Heart Institute, Hadassah Hebrew University Medical Center, Jerusalem 91200, Israel; etty.grad@mail.huji.ac.il (E.G.); haimd@ekmd.huji.ac.il (H.D.D.); 5 Allergy and Clinical Immunology Unit and Department of Medicine, Hadassah University Medical Center, Jerusalem 91200, Israel; talyuv@gmail.com; 6 Division of Cardiovascular Medicine, Beth Israel Deaconess Medical Center, Harvard Medical School, Boston, MA 02115, USA; pkang@bidmc.harvard.edu; 7 Department of Ophthalmology, Harvard Medical School, Boston, MA 02115, USA

**Keywords:** myocardial infarction, apoptosis, angiogenesis, PR1P, prominin-1, VEGF

## Abstract

Myocardial infarction (MI) remains the leading cause of death in the western world. Despite advancements in interventional revascularization technologies, many patients are not candidates for them due to comorbidities or lack of local resources. Non-invasive approaches to accelerate revascularization within ischemic tissues through angiogenesis by providing Vascular Endothelial Growth Factor (VEGF) in protein or gene form has been effective in animal models but not in humans likely due to its short half-life and systemic toxicity. Here, we tested the hypothesis that PR1P, a small VEGF binding peptide that we developed, which stabilizes and upregulates endogenous VEGF, could be used to improve outcome from MI in rodents. To test this hypothesis, we induced MI in mice and rats via left coronary artery ligation and then treated animals with every other day intraperitoneal PR1P or scrambled peptide for 14 days. Hemodynamic monitoring and echocardiography in mice and echocardiography in rats at 14 days showed PR1P significantly improved multiple functional markers of heart function, including stroke volume and cardiac output. Furthermore, molecular biology and histological analyses of tissue samples showed that systemic PR1P targeted, stabilized and upregulated endogenous VEGF within ischemic myocardium. We conclude that PR1P is a potential non-invasive candidate therapeutic for MI.

## 1. Introduction

Myocardial infarction (MI) is a leading cause of morbidity and mortality worldwide [1]. Rescuing cardiomyocytes (CM) from cell death in the ischemic region and restoring blood flow in obstructed arteries are two crucial strategies for treatment. Conventional invasive therapies to improve coronary artery blood flow and reduce myocardial damage following MI include coronary artery dilation or stenting and coronary artery bypass surgery. However, these invasive procedures are often not available, require delay (often due to comorbidities) or are not entirely effective. Therapeutic induction of angiogenesis would potentially be more easily available and less invasive, as it could theoretically only require the systemic administration of angiogenic factors to restore vascularization to damaged areas of the heart. The most promising and extensively studied pro-angiogenic therapeutic is vascular endothelial growth factor (VEGF) [2], which has been shown to function as both a survival and angiogenesis factor [3]. Specifically, VEGF facilitates CM regeneration and protects CMs from apoptosis in vitro by activation of downstream VEGF signaling pathways including the activation of AKT and the upregulation of Bcl-2 expression [4,5]. However, despite success in animal ischemia models, VEGF therapies have failed in human clinical trials [6]. Disappointing clinical results can potentially be attributed to short plasma and tissue half-lives, which limit the retention of VEGF within target (ischemic) zones. Additionally, VEGF’s propensity to induce severe hypotension and tissue edema when delivered intravascularly limited dosing to levels far below those likely to show efficacy in under perfused areas [7,8].

We have previously shown that PR1P, a small peptide derived from the extracellular domain of prominin-1 and which binds to VEGF and protects it from proteolytic degradation [9,10], augmented angiogenesis in multiple in vivo angiogenesis models and improved perfusion of threatened limbs in a murine hind limb ischemia model [9]. Additionally, PR1P has been shown to increase VEGF levels and signaling within lungs when delivered via inhalation [10] and to protect neurons [11] and lung cells [10] from apoptosis. We hypothesized here that PR1P would upregulate endogenous VEGF signaling within compromised heart tissue and improve outcome from myocardial infarction. Herein, we provide evidence that PR1P improved CM survival following serum starvation in vitro, and when delivered systemically to mice and rats following left coronary artery ligation, PR1P-targeted stabilized and upregulated VEGF signaling within ischemic myocardium. This targeted upregulation of endogenous VEGF was associated with improved functional outcome measured using intravascular hemodynamic monitoring and echocardiography. Collectively, these data support using PR1P in a novel non-invasive approach to treat MI by targeting and upregulating endogenous VEGF within ischemic myocardium.

## 2. Results

### 2.1. PR1P Improves Murine Myocardial Function Post-Infarction

**Figure 1:**
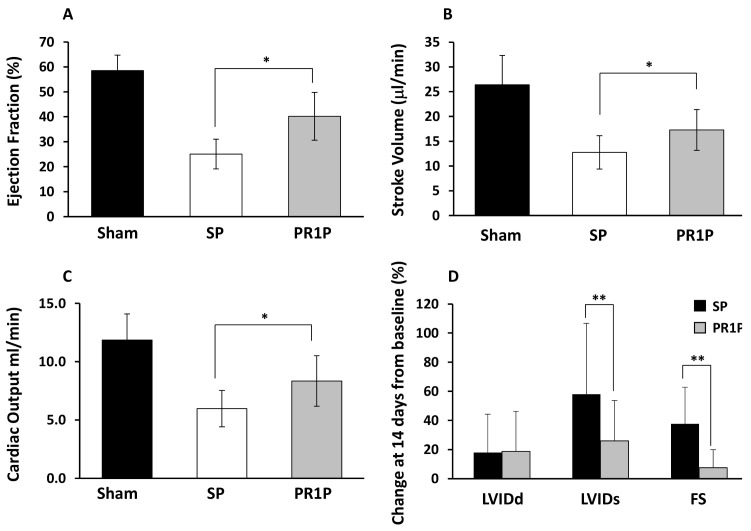
PR1P enhances the functional outcome of the heart following myocardial infarction in mice. (**A**–**C**) Bar graphs showing left ventricular ejection fraction (EF, **A**) stroke volume (SV, **B**) and cardiac output (CO, **C**) measured using left ventricular vascular transducer catheters in anesthetized mice 14 days following sham surgery (Sham) and no treatment, or left coronary artery ligation and every other day intraperitoneal injections with scrambled peptide (SP) or PR1P. *n* = 6 SHAM, 17 SP, 18 PR1P. * *p* < 0.006 using ANOVA and Tukey post hoc analysis comparing PR1P vs. SP. (**D**) Bar graph showing average relative change from baseline at 14 days of indicated echocardiographic measurements made on anesthetized mice similarly treated as described in Figure 1A indicating improved outcome following PR1P treatment. Left ventricular internal diameter at end diastole (LVIDd), left ventricular internal diameter at end systole (LVIDs) and fractional shortening (FS) calculated as FS = ((LVIDd − LVIDs)/LVIDd). *n* = 7 SP, 11 PR1P. ** *p* < 0.015 using Student’s t-test.

To determine whether systemic PR1P therapy could be used to improve the functional outcome of injured myocardial tissue in vivo, we used invasive and non-invasive tools to assess myocardial performance at 14 days post surgically induced MI in mouse and rat MI models. In these models, surgical ligation of the left anterior descending coronary artery leads to myocardial infarction characterized initially by increased levels of VEGF mRNA and protein expression in compromised myocardium [12,13,14,15]. There was no mortality from surgery, and no differences between groups in body weights before, during or after experiments in any of the animals. As summarized in Table 1, PR1P treatment in mice led to significant improvement compared to SP in maximum left ventricular volume (MAX-LV-V, 45.4 ± 7.3 in PR1P, 53.2 ± 6.7 in SP) and in the minimum left ventricular volume (MIN-LV-V, 22.8 ± 6.8 in PR1P, 35.5 ± 6.5 in SP) at 14 days post coronary artery ligation as determined using left ventricular vascular catheter probes. In addition, PR1P treatment led to improved stroke volume (17.3 ± 4.1 in PR1P, 12.8 ± 3.4 in SP) and stroke work (1493 ± 414 in PR1P, 1066 ± 296 in SP) compared to SP similarly assessed using intraventricular probes at 14 days. Furthermore, PR1P improved flow-based hemodynamic cardiac function parameters including ejection fraction (EF, Figure 1A), stroke volume (SV, Figure 1B) and cardiac output (CO, Figure 1C). Echocardiography revealed that PR1P reduced left ventricular internal diameter at end systole (i.e., LVIDs, Figure 1D) and mitigated the reduction in left ventricular fractional shortening (FS, Figure 1D) at day 14 following coronary artery ligation. Collectively, these data demonstrate that systemic PR1P therapy significantly improved outcome from coronary artery ligation in mice.

### 2.2. PR1P Improves Rat Myocardial Function Post-Infarction

**Figure 2:**
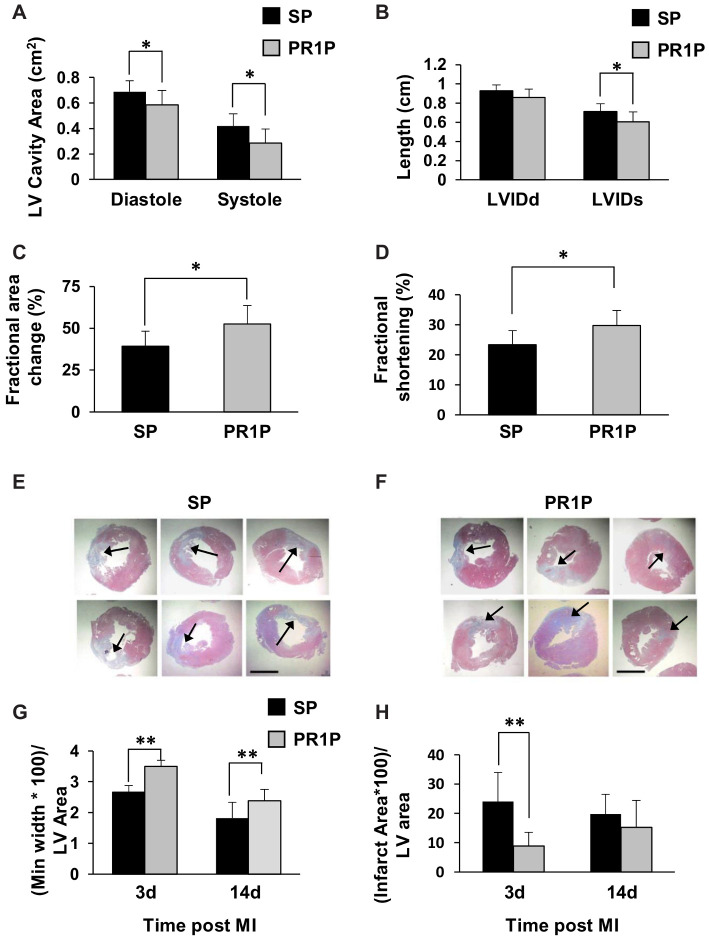
PR1P enhances the functional outcome of the heart following myocardial infarction in rats. (**A**–**D**) Bar graphs showing echocardiographic assessments of rat heart function at 14 days following left anterior coronary artery ligation and every other day treatment with PR1P or scrambled peptide (SP) indicating improved outcome following PR1P treatment. Left ventricular cavity area (LV cavity area), left ventricular internal diameter at end diastole (LVIDd), left ventricular internal diameter at end systole (LVIDs) and fractional shortening (FS). (*n* = 9 PRIP, 7 SP) * *p* < 0.04, (**E**–**H**) PR1P reduces infarct size following ischemia. (**E**,**F**) Representative photomicrographs of Masson trichrome stained sections of rat heart from animals described in Figure 2A indicating infarcted regions (collagen, blue) and regions with viable muscle (pink red) at 14 days after surgery. Black arrows indicate areas of infarction. Scale bar, 100 mm. (**G**,**H**) Bar graphs showing quantification of infarct size from photomicrographs described in E–F from hearts prepared 3 (*n* = 5 PR1P, 5 SP) and 14 (*n* = 6 PR1P, 5 SP) days after surgery. ** *p* < 0.05.

We next characterized the ability of systemic PR1P to mediate functional outcome from myocardial infarction in rats. In these studies, echocardiography at two weeks post coronary artery ligation demonstrated decreased LV dilatation post-MI as evidenced by a reduction in the left ventricular cavity area during both systole and diastole (LV Cavity Area, Figure 2A) and in the LVID at the end of systole (LVIDs) and diastole (LVIDd, Figure 2B). In addition, PR1P therapy led to improved cardiac contractility as indicated by an increase in the fractional change in area (Figure 2C) and increased left ventricular shortening fraction (Figure 2D). Representative photomicrographs of Masson trichrome stained cross sections of myocardium (to identify collagen at sites of infarction [16]) from PR1P- and SP-treated rats 14 days after coronary artery ligation surgery are shown in Figure 2E–F. Quantification of the infarct sizes from micrographs in similarly stained heart sections show decreased areas of infarction at both 3 and 14 days from surgery (Figure 2G–H). Taken together, these data suggest that systemic delivery of PR1P enhances the functional outcome of murine and rat myocardium following acute ischemic injury.

### 2.3. PR1P Stabilizes VEGF, Upregulates VEGF Signaling and Mitigates Apoptosis

**Figure 3:**
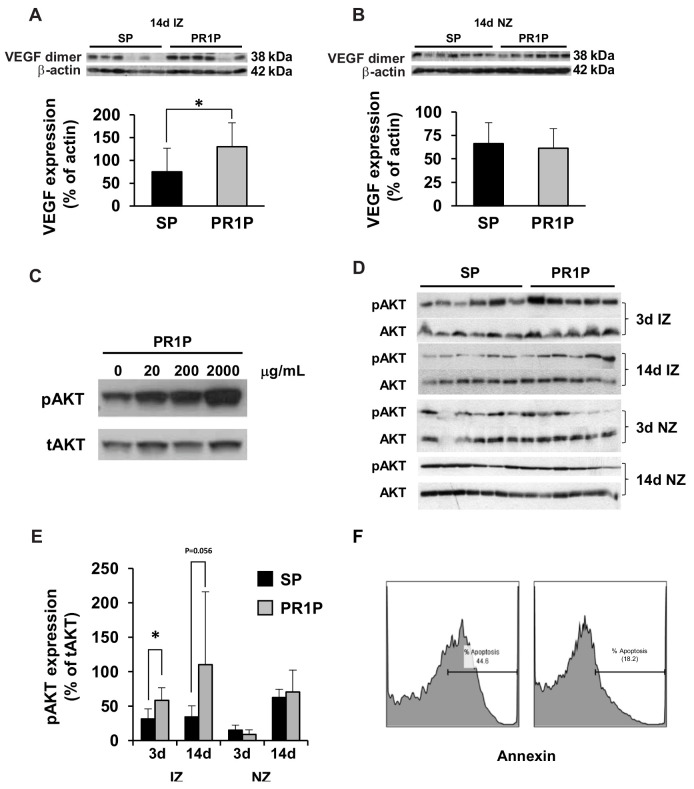
PR1P targets, stabilizes and upregulates endogenous VEGF within ischemic myocardium. (**A**,**B**) Representative Western blot analyses (at top) and corresponding bar graphs showing quantification of blots of rat heart tissue homogenates from ischemic zone (IZ, **A**) or normal zone (NZ, **B**) at 14 days following left coronary artery ligation and every other day intraperitoneal treatment with PR1P or scrambled peptide (SP). Bar graphs show the corresponding quantification of VEGF expression as a percentage of β-actin expression for indicated groups of myocardial tissue homogenates and indicate that PR1P treatment augments the expression of VEGF in the IZ. * *p* < 0.05. (**C**) Representative Western blot analysis of protein from human cardiomyocyte cell homogenates following 15-minute incubation in vitro at 37 °C in serum free cell culture media in the presence of increasing concentrations of PR1P showing dose-dependent increase in phosphorylated AKT (pAKT) relative to total AKT (tAKT). (**D**) Representative Western blot analyses (**D**) and corresponding bar graphs (**E**) showing quantification of phosphorylated AKT (p-AKT) relative to AKT (total, t-AKT) of rat heart tissue homogenates from ischemic or normal zones (IZ or NZ, respectively) at 3 or 14 days following left coronary artery ligation and treatments described in Figure 3A. Membranes were immunoblotted with antibodies to phosphorylated AKT (p-AKT) and then AKT (total, t-AKT) in order to quantify the relative expression of pAKT as a percentage of tAKT. (**E**) Bar graphs showing corresponding quantifications of pAKT expression as a percentage of tAKT in IZ and NZ myocardium at indicated time points showing increased pAKT in IZ following PR1P therapy. * *p* < 0.01. (**F**) Representative FACS analysis of cardiomyocytes following 48-hour serum starvation in the presence or absence of PR1P (0.2 mg/mL) and stained with FITC-annexin V showing that PR1P reduces serum starvation-induced apoptosis.

We next turned to molecular biology studies to determine the molecular mechanism by which PR1P augments recovery from myocardial ischemia caused by coronary artery ligation. We recently showed that PR1P bound VEGF, enhanced the prevalence of VEGF dimers (the active form of VEGF) and prevented VEGF degradation in vitro and in vivo in the lungs when delivered via inhalation [10]. We, therefore, investigated here whether systemically delivered PR1P similarly stabilized VEGF dimers within injured myocardium. Western blot analyses of tissue homogenates from ischemic and non-ischemic zones from rat heart biopsies 14 days after coronary artery ligation and every other day treatment with PR1P or SP showed increased expression of VEGF dimers in ischemic zones in the presence of PR1P (IZ, Figure 3A). Interestingly, there was no effect of PR1P on the prevalence of VEGF dimers in non-ischemic zones (NZ, Figure 3B) in this same time period.

We previously showed that PR1P significantly reduced retinal ganglion cell apoptosis in an optic nerve crush injury model [11] and prevented respiratory epithelial cell apoptosis from cigarette smoke extract in vitro and from LPS in vivo in mice [10]. Downstream VEGF signaling including the phosphorylation and activation of the protein kinase AKT has been shown to inhibit CM apoptosis [17,18,19]. Akt also plays a key role in multiple cellular processes that mediate cell survival during stress including glucose metabolism, gene transcription, cell proliferation and cell migration [20,21]. To determine whether PR1P mediates AKT phosphorylation and apoptosis in injured myocardial cells, we first incubated CMs in vitro in the presence of increasing concentrations of PR1P and assessed levels of phosphorylated AKT in cell homogenates using Western blotting. Figure 3C shows that PR1P increased AKT phosphorylation in these cells at 15 min in a dose-dependent manner. To determine whether PR1P similarly augments AKT phosphorylation in vivo following tissue injury, we biopsied cardiac tissue from the ischemic zone (IZ) and normal zone (NZ) of rat heart at 3 and 14 days following coronary artery ligation and analyzed levels of phosphorylated AKT relative to total AKT in tissue homogenates (Figure 3D,E). Quantification of Western blots in these experiments revealed that PR1P increased AKT phosphorylation at 14 days following MI in the ischemic zones only (Figure 3E). Together with our findings that PR1P augmented levels of VEGF dimers in ischemic zones only, these results suggest that PR1P targets and stabilizes endogenous VEGF and augments VEGF signaling only within sites of ischemic myocardium. To determine whether PR1P has the potential to mediate apoptosis in CMs, we cultured CMs in serum-free medium for 48 h in the presence and absence of PR1P and then fixed and stained the cells with annexin V (to identify cell apoptosis). FACS analysis of these cells revealed that PR1P significantly reduced serum starvation-induced CM apoptosis (Figure 3F). Together, these data strongly suggest that systemic PR1P therapy augmented the functional outcome from coronary artery ligation by targeting and upregulating endogenous VEGF signaling within ischemic myocardium.

## 3. Discussion

Here, we report on studies conducted in mice and rats supporting that PR1P is a novel candidate therapeutic to treat MI. We used established rodent cardiac ischemia models involving young female animals that we and others have used successfully [22,23,24,25]. Although biological sex and relative estrogen levels have been shown to be protective against cardiovascular disease in rodent models [26], the rapid recovery from injury seen in female rodents following acute ischemia provides a model in which to evaluate for therapeutic efficacy within an active healing and remodeling process. Acute MI in humans and in rodents is accompanied by transient upregulation of VEGF expression and signaling following injury [13,14,15] and, thus, a likely target environment for PR1P, which binds, stabilizes and upregulates endogenous VEGF [10]. Although no single coronary artery disease animal model satisfies all clinical conditions required to unequivocally prove that experimental drugs may be used in human disease [27], we found that systemic delivery of PR1P resulted in improved cardiac function two weeks from coronary artery ligation, as measured by echocardiography and invasive intravascular monitoring. Mechanistic studies suggested that systemically delivered PR1P targeted cardiac tissue within ischemic zones leading to sustained upregulation of endogenous VEGF signaling beyond that which is normally found during natural recovery from MI in rodents.

Myocardial infarction affects 7.9 million US adults annually [28]. As populations age, an increasing proportion of patients who might benefit from standard revascularization procedures will be rejected as candidates due to significant comorbidities [29]. Thus, there has been an interest to develop an effective and easily accessible non-invasive treatment capable of reducing myocardial injury following ischemia. Gene, protein and cellular therapies have been considered as potential strategies to treat coronary artery and peripheral vascular occlusive diseases that do not respond to conventional treatment [30]. VEGF remains the leading candidate target to stimulate both angiogenesis and prevent apoptosis, the two major processes that mediate atherosclerosis as well as ischemic heart disease [31]. However, although different types of VEGF therapies, including treatment with genes and proteins, have been shown to be effective in animal myocardial infarction and ischemic limb models, clinical trials for these purposes have not been successful [32,33]. Failure in these studies was multi-factorial. VEGF is rapidly cleared from the circulation [14,34], and its tissue half-life is short [35], and so high VEGF doses required to prove therapeutic efficacy were used, which resulted in significant toxicity including hypotension, localized edema, anemia and thrombocytopenia [36,37]. Myocardial VEGF production is significantly but only transiently upregulated under normal circumstances following ischemic injury [12]. It would, therefore, seem plausible that a therapeutic such as PR1P that can target, stabilize and upregulate endogenous VEGF signaling within tissue microenvironments for extended periods of time without disturbing pre-existing VEGF gradients may prove beneficial in mitigating tissue injury and may also limit systemic toxicity. Targeted catheter-mediated intracoronary VEGF-A gene transfer was shown to be safe and did not increase the risk of cardiovascular or systemic toxicity during 10 year follow up [38,39]. Importantly, treatment with PR1P is not akin to VEGF therapy, as it does not expose tissue to exogenous VEGF or inappropriate VEGF gradients. PR1P addresses the need for an alternative systemic therapy that targets and enhances the effect of endogenous VEGF only within sites of tissue injury (see Figure 3). Although this study was not designed to measure toxicity, unpublished data suggest a lack of toxicity in mice and rats exposed to daily high doses of PR1P (100-fold higher than the treatment dose) for two weeks. It will be necessary to evaluate for potential PR1P toxicity during drug development should the peptide prove therapeutic in larger animal studies. Unlike with systemic VEGF therapy, the hypoxia-induced VEGF gradient in the injured tissue microenvironment is targeted by PR1P and is maintained, thereby preserving the mechanical and chemical cues necessary to stimulate appropriate blood vessel growth and myocardial repair [40,41].

The role of VEGF signaling following ischemia in the rodent heart after coronary artery ligation has been extensively studied [31,42]. Within hours of coronary artery ligation, there is a transient increase in the level of VEGF mRNA in zones bordering ischemic regions that returns to baseline within 24 h [14]. In contrast, there is a gradual decline in VEGF mRNA in ischemic zones that begins hours after ischemia and that remains low 2 weeks thereafter [14]. VEGF levels similarly increase transiently for 24 h in border zones, and similarly decline within hours in ischemic zones and remain low for up to one month [14]. Interestingly, there is inflammation within the injured myocardium that becomes apparent within hours to days: neutrophils infiltrate the ischemic tissue within the first few days, then macrophages appear (~4 days) followed by lymphocytes (7–14 days) [42]. There is a proliferation of fibroblasts and collagen accumulation early beginning within the 7–14 days, and there is completion of scar formation by day 21 [42]. Thus, ischemia induced VEGF signaling within the border and ischemic zones mediates angiogenesis and multiple cellular events including apoptosis and inflammation that ultimately determine the degree of functional recovery of the myocardium [12,14]. We found that every other day treatment with systemic PR1P both targets and stabilizes endogenous VEGF within ischemic zones and alters the natural progression of endogenous VEGF signaling that was associated with improved functional outcome. Specifically, PR1P therapy led to an increase in VEGF dimer levels and increased downstream VEGF signaling (AKT phosphorylation) within ischemic zones, but not within zones remote from injury (see Figure 3). Our study is limited by the fact that we currently lack the technology to measure PR1P concentration in blood or tissues, in particular in ischemic and remote ischemic zones. Such an ability might reveal the mechanism by which PR1P targets endogenous VEGF within ischemic tissue. We recently showed that PR1P binding to VEGF prevented its degradation by proteases, including plasmin and elastase, naturally released by inflammatory cells during inflammation, and which compete with PR1P for binding sites within the VEGF heparin binding domain (HBD) [10]. PR1P binding to VEGF within ischemic zones of the myocardium likely similarly increases endogenous VEGF levels and signaling by preventing VEGF degradation by proteases released by inflammatory cells recruited into the heart following ischemia. Ohta et al. found that increased elastase activity that correlated with inflammatory cell infiltration of the murine heart following MI increased as early as 6 h after left coronary artery ligation and persisted at 7 days [43]. Overexpression of an elastase inhibitor in mice in their model led to an improvement in diastolic dysfunction at 4 days and improved cardiac performance at 28 days following coronary artery ligation, suggesting that elastase inhibition suppresses inflammation associated cardiac dilatation and dysfunction after MI [43]. Importantly, PR1P does not inhibit elastase per se but instead blocks its binding to VEGF [10]. As such, the effect of PR1P on the functional outcome in the ischemic myocardium may be due in part to its ability to prevent VEGF degradation by elastase and other proteases, which might limit potential signaling effects of VEGF degradation products [44,45]. Kurtagic et al. showed that VEGF degradation product generated by elastase is a macrophage chemoattract [45], and so reduced VEGF degradation product could in itself mitigate inflammation. Although the mechanism by which PR1P targets the injured myocardium is not clear, this novel property of targeting VEGF signaling where needed could potentially be used to target oxygen-deprived but not yet infarcted regions in the heart during unstable angina or for peripheral vascular occlusive disease where inflammation is also noted [46,47].

Our studies revealed that despite improvement in the function of the rat myocardium following every other day treatment with PR1P for 14 days following coronary artery ligation, there was no significant difference in the size of infarcts between the groups (Figure 2A–D). One limitation of the rat study is that echocardiography was not done on animals at baseline following LAD ligation to ensure equal deleterious effects on cardiac function between groups. Nevertheless, our results highlight the importance of the cardiac remodeling process that follows ischemia and suggest that despite the size of the infarct, the quality of remodeling can be a significant variable that affects outcome. Interestingly, VEGF is directly or indirectly implicated in nearly all of the key processes that mediate remodeling including apoptosis, energy metabolism, oxidative stress, inflammation, collagen deposition, alterations in contractile proteins, calcium transport, geometry and neuro-hormonal activation [31,48,49,50,51,52,53,54,55]. Thus, despite equivalent sized infarcts, our data suggest that PR1P-induced changes in local VEGF signaling within the ischemic myocardium that mediated the remodeling process in a way that improved global myocardial function. Further work to delineate how PR1P may affect the cardiac remodeling process is warranted.

We found that PR1P stabilized VEGF in its dimeric (active) form within ischemic zones of rat myocardium at 2 weeks following coronary artery ligation leading to upregulation of endogenous VEGF signaling. Downstream VEGF signaling has been shown to play a key role in multiple cellular processes in CMs that mediate cell survival during stress including glucose metabolism, gene transcription, cell proliferation and cell migration [20,21]. In addition, VEGF-mediated AKt activation has been shown to reduce apoptosis in CMs following ischemia-reperfusion injury [56,57], pressure overload [58] and oxidative stress [59]. Thus, these multiple cellular processes collectively mediate cell survival, along with angiogenesis, which would serve to augment perfusion to deprived tissues and ultimately determine the functional outcome of the heart following ischemic injury in vivo [51]. In fact, increased expression of anti-apoptotic proteins in cardiomyocytes, such as Bcl-2, and increased apoptosis in general is a consistent feature of end-stage heart failure [60] and correlates with the clinical severity of cardiomyopathy [5]. Interestingly, knockdown of Akt resulted in impaired endothelial progenitor cell function and neovascularization of hind-limb muscles following experimentally induced unilateral limb ischemia in mice [61]. PR1P upregulated angiogenesis in multiple VEGF-dependent angiogenesis models in vitro and in vivo and in so doing increased blood flow to compromised hind limbs in a murine hind-limb ischemia model [9]. Our current study further supports that PR1P therapy enhances outcomes following tissue injury by targeting endogenous VEGF in the ischemic tissue where it facilitates AKt phosphorylation and the downregulation of apoptosis.

In summary, these studies revealed that systemically administered PR1P targeted and stabilized VEGF to augment VEGF levels and signaling in ischemic zones following coronary artery ligation in mouse and rat MI models. Sustained stabilization and upregulation of endogenous VEGF led to improved functional outcome of the heart in both animal models at 2 weeks from MI. Our findings have direct relevance to human diseases involving tissue injury from arterial insufficiency leading to tissue ischemia including coronary artery and peripheral vascular disease. Success of this novel VEGF targeting approach would enable patients to be treated earlier and without the need for invasive technologies.

## 4. Material and Methods

### 4.1. Rodent Models of Myocardial Infarction

Ten-week-old female C57BL/6J mice (Jackson Laboratory (Bar Harbor, ME and from Harlan, Rehovot, Israel) and 10-week-old female Sprague-Dawley rats (Harlan, Rehovot, Israel) were used for the MI experiments. Anesthesia was induced using ketamine hydrochloride (80 mg/kg) and xylazine hydrochloride (5 mg/kg). Upon adequate anesthesia, a left thoracotomy was performed, the heart was exposed, and the left anterior descending coronary artery ligated using a 6.0 silk suture. Following ligation, the thorax and skin were closed, and the animals allowed to recover at ambient room temperature. Upon full recovery from anesthesia (i.e., within 1–2 h) animals were randomized in a blinded fashion to receive treatment consisting of intraperitoneal (IP) administration of PR1P or scrambled peptide (SP, 10 µg in 100 µL saline/mouse and 200 µg in 100 µL saline/rat), starting on day 0 (i.e., after surgery) and administered every other day until day 12. A select group of animals underwent Sham surgery and did not receive treatment of any kind. Animals were euthanized on day 14. Protocols for these studies were approved by the Institutional Animal Care and Use Committee (IACUC) at Boston Children’s Hospital (Ethics Committee - research number: 09071484: Approval Date: 19 April 2010. Duration: From 19 April 2010 to 19 April 2013) and at the Beth Israel Deaconess Medical Center (BIDMC) (murine MI model Ethics Committee - research number: 084-2010: Approval Date: 21 September 2010 Duration: From 21 September 2010 to 21 September 2013) and Institutional Animal Care and Use Committee (IACUC) at the Hebrew University, Jerusalem, Israel (rat MI model: Ethics Committee - research number: MD-12-13366-4 Approval Date: 16 May 2012 Duration: From 22 May 2012 to 22 May 2015).

### 4.2. Hemodynamic Measurements in Mice

Hemodynamic pressure-volume parameters were measured in mice under inhaled isoflurane anesthesia using a 1.4 Fr microtip pressure-volume catheter (Scisense, London, ON, Canada) inserted into the right common carotid artery and advanced into the left ventricle. Data were recorded using a Powerlab system (ADInstruments, Colorado Springs, CO, USA). Beat-by-beat pressure-volume parameters including stroke work (SW), cardiac output (CO) and ejection fraction (EF) were measured and analyzed using CardioSoft Pro software, as was done in the past (CardioSoft, Houston, TX, USA) [62,63]. Maximum left ventricular pressure (MAX-LVP), minimum left ventricular pressure (MIN-LVP), minimum end diastolic left ventricular pressure (EDM-LVP), maximum left ventricular volume (MAX-LV-V) and minimum left ventricular volume (MIN-LV-V).

### 4.3. Echocardiographic Studies in Mice and Rats

Echocardiography was performed in mice and rats by a blinded technician on anesthetized ((ketamine, 80 mg/kg, IP) and xylazine hydrochloride (5 mg/kg, IP)) animals at 3 or 14 days after coronary artery ligation surgery. Echocardiographic imaging was performed using a GE Vivid3 platform equipped with a 13-MHz linear epiaortic transducer (General Electric, Haifa, Israel). The probe was positioned in a left parasternal position, and two-dimensional imaging of the heart in the short axis was performed using a high frame rate. This image was used to guide an M-mode cursor down the medial axis of the LV. Measurements were performed in triplicate using the leading-edge convention for myocardial borders.

### 4.4. Histology and Immunochemistry (Rat Heart Infarct Quantification)

Rat hearts were fixed in 10% formalin and paraffin-embedded using standard protocols. Heart sections (4 μm) were treated with Masson’s trichrome (MTC, Thermo Scientific, Rockford, IL, USA), which stains collagen blue and myocardial cells pink red. Photomicrographs of sections of the left ventricle were used to quantify myocardial infarction size as described by Grad et al. [16]. Briefly, infarct size was calculated as a normalized ratio of the minimal width of the infarcted area compared to the area of the left ventricle (LV) muscle area ((min width × 100)/LV Area, i.e., blue stain)/total LV muscle area (cm^2^), as well as a normalized ratio of the infarct area to the whole left ventricle area ((Infarct Area × 100)/LV area) using ImageJ© software (National Institute of Health, Bethesda, MD, USA).

### 4.5. Cell Culture

Primary human cardiomyocytes (HCM) derived from human adult heart were purchased from ScienCell Research Laboratories (Carlsbad, CA, USA). The cells were grown in Cellartis CM Culture base (Takara, Mountain View, CA, USA) and used between passages 4–6 [64].

### 4.6. Apoptosis Assay

Cardiomyocytes (CMs) were incubated in serum free medium without supplements in the presence or absence of PR1P (0.2 mg/mL) for 48 h, removed from the plate by treatment with 0.25% trypsin and then incubated with FITC-conjugated Annexin V to identify apoptotic cells (Ebioscience, San-Diego, CA, USA) according to manufacturer’s instructions. Cells were then analyzed on FACS Calibur flow cytometer (BD Biosciences, Pharmingen, San Diego, CA, USA) using Flowjo software.

### 4.7. Western Blot Analysis

CMs were serum starved for 48 h and incubated with 50–2000 μg/mL of PR1P for 15 min at 37 °C. The cells were scraped and washed twice with 1× PBS. Cells were homogenized with RIPA buffer [50 mMTris-HCl (pH 7.4), 150 mMNaCl, 1% Nonidet P-40, 0.5% Na+ deoxycholate and 0.1% SDS] (IPA-Boston Bioproducts, Worchester, MA, USA), containing a protease inhibitor cocktail tablet (Sigma-Aldrich, St. Louis, MO, USA) on ice. Blotting was done using standard methods and repeated three times to ensure reproducibility. Primary antibodies for Western blot analysis in this study were used at 1:1000 dilution and included anti-VEGF (Santa Cruz, Santa Cruz, CA, USA), pAKT (Cell signaling, Danvers, MA, USA) and β-actin (Sigma-Aldrich, St. Louis, MO, USA).

### 4.8. Reagents and Peptides

The 12-mer peptide PR1P (DRVQRQTTTVVA) and SP (QATVDTRQVTRV) were commercially synthesized by Biomatik (Wilmington, DE, USA).

### 4.9. Statistical Analysis

All results are expressed as mean ± SD. Statistical comparisons between multiple groups were performed using ANOVA and Tukey’s post hoc test (multiple comparisons test). Prior to analyses using ANOVA all datasets were confirmed to have normal distribution using the D’Agostino and Pearson normality test. Equal variance in these datasets were confirmed using the Bartlett’s Test. Statistical comparisons between two groups were performed by Student’s t-test. Prior to analyses using Student’s t-test, all datasets were confirmed to have normal distribution using the D’Agostino–Pearson omnibus (K2) test. Equal variance in these datasets were confirmed using the F-test. In conditions of unequal variance, Student’s t-test was performed using the Welch’s correction. *p* values < 0.05 were considered statistically significant. Statistical testing was performed using Prism 8 for MacOS version 8.2.2 (159) (GraphPad Software, San Diego, CA, USA). All experiments were repeated in triplicate.

## Figures and Tables

**Table 1:** PR1P enhances functional outcome of the heart following myocardial infarction in mice. Shown are hemodynamic parameters and indices of heart function (with *p* values comparing groups as indicated) in mice obtained using vascular transducer catheters placed in the left ventricle of anesthetized animals 14 days following sham surgery (SHAM) and no treatment, or left coronary artery ligation and every other day treatments with intraperitoneal injections of PR1P or scrambled peptide (SP). Beats per minute (BPM), maximum left ventricular pressure (MAX-LVP), minimum left ventricular pressure (MIN-LVP), minimum end diastolic left ventricular pressure (EDM-LVP), maximum left ventricular volume (MAX-LV-V) and minimum left ventricular volume (MIN-LV-V). *n* = 6 SHAM, 17 SP, 18 PR1P.

	**Heart Rate (bpm)**	**MAX-LVP (mmHg)**	**MIN-LVP (mmHg)**	**EDM-LVP (mmHg)**	**MAX-LV_V (uL)**	**MIN-LV_V (uL)**
SHAM	452.8 ± 22.8	100.9 ± 4.9	−3.4 ± 2	1.9 ± 0.6	49.2 ± 11.2	15.8 ± 5.9
SP	468.9 ± 48.3	89.7 ± 6.3	7.9 ± 5.2	16 ± 6.4	53.2 ± 6.7	35.5 ± 6.5
PR1P	483.5 ± 66.8	94.5 ± 8.3	5 ± 6.6	10.7 ± 7.6	45.4 ± 7.3	22.8 ± 6.8
SHAM vs. SP	0.8149	**0.0052**	**0.0005**	**0.0002**	0.538	**0.0001**
SHAM vs. PR1P	0.4778	0.1433	**0.009**	**0.0203**	0.5633	0.0986
PR1P vs. SP	0.7215	0.1258	0.2954	0.0600	**0.0151**	**0.0001**
	**Stroke Volume (uL)**	**Stroke Work** **(mjoules)**	**dp/dt MAX** **(mmHg/Sec)**	**dp/dt MIN** **(mmHg/Sec)**	**Aortic Pressure MAX Systolic (mmHg/Sec)**	**Aortic Pressure MIN Diastolic** **(mmHg/Sec)**
SHAM	26.4 ± 5.9	2672 ± 719	8677 ± 751	−7876 ± 919	100.2 ± 5.1	70.8 ± 7.5
SP	12.8 ± 3.4	1066 ± 296	5999.7 ± 1031	−4942 ± 765	90.2 ± 5.6	68.6 ± 5.5
PR1P	17.3 ± 4.1	1493 ± 414	6817.7 ± 1518	5214.1 ± 1050	92.9 ± 7.9	71.9 ± 5.7
SHAM vs. SP	**0.0001**	**0.0001**	**0.0002**	**0.0001**	**0.0073**	0.7283
SHAM vs. PR1P	**0.0001**	**0.0001**	**0.0078**	**0.0001**	**0.0586**	0.9120
PR1P vs. SP	**0.0061**	**0.0159**	0.1372	0.6534	0.4489	0.2415
